# Mental health approach to premenstrual dysphoric syndrome: case series

**DOI:** 10.1192/j.eurpsy.2025.2413

**Published:** 2025-08-26

**Authors:** A. Izquierdo De La Puente, P. Del Sol Calderon, R. Fernandez Fernandez

**Affiliations:** 1 Psiquiatria, Hospital Universitario Puerta de Hierro de Majadahonda; 2 Psiquiatria, Hospital Universitario Infanta Cristina, Madrid, Spain

## Abstract

**Introduction:**

Six clinical cases of patients with premenstrual dysphoric syndrome treated in Mental Health with SSRIs are presented.

**Objectives:**

The aim is to briefly review the pharmacological approach to premenstrual dysphoric syndrome through the presentation of a series of cases.

**Methods:**

Six cases of female patients are presented, with a mean age of 35.4 years, two of whom were nulliparous. All of them had no history of mental health problems. They had regular cycles and had no relevant medical or gynecological history. They reported that for the last three years they had been more irritable, emotionally labile, feeling apathetic and sad, with difficulties in concentration and a feeling of loss of self-control, which made interpersonal relationships difficult, especially at work.

The patients denied that they had previously experienced these symptoms throughout their lives.

Analyses were carried out, with estrogen and progesterone levels, without obtaining significant alterations.

The MADRS and HAMILTON scales were administered to all of them on the 14th day of the cycle, as well as on the 5th day of the cycle. A mean of 9.2 was obtained on the MADRS on the 5th day of the cycle, compared to 15.6 on the 14th day, while the HAMILTON obtained a score of null-mild anxiety on the 5th day and moderate anxiety on the 14th day.

**Results:**

After this comparison, treatment with fluoxetine at a dose of 20mg DMD was started only from the day of ovulation to menstruation, withdrawing this treatment for the rest of the cycle. Again, both scales were compared and the results obtained were more similar on the 5th and 14th day of the cycle.

**Conclusions:**

To avoid hormonal treatment and thus the moderate side effects it presents, premenstrual dysphoric syndrome can be treated by taking SSRIs at low doses, only for 15 days of the cycle.

**Disclosure of Interest:**

None Declared

